# The tRNA-derived fragment 5026a inhibits the proliferation of gastric cancer cells by regulating the PTEN/PI3K/AKT signaling pathway

**DOI:** 10.1186/s13287-021-02497-1

**Published:** 2021-07-22

**Authors:** Linwen Zhu, Zhe Li, Xiuchong Yu, Yao Ruan, Yijing Shen, Yongfu Shao, Xinjun Zhang, Guoliang Ye, Junming Guo

**Affiliations:** 1 Department of Gastroenterology, The Affiliated Hospital of Ningbo University School of Medicine, Ningbo, 315020 China; 2 Department of Biochemistry and Molecular Biology, and Zhejiang Key Laboratory of Pathophysiology, Medical School of Ningbo University, Ningbo, 315211 China; 3 Affiliated Lihuili Hospital of Ningbo University, Ningbo, Zhejiang 315041 China; 4 Institute of Digestive Diseases of Ningbo University, Ningbo, 315020 China

**Keywords:** tRNA-derived small RNAs, tRF-5026a, tRF-18-79MP9P04, Gastric cancer, PTEN, PI3K

## Abstract

**Background:**

Recently, tRNA-derived fragments (tRFs) have been shown to serve important biological functions. However, the role of tRFs in gastric cancer has not been fully elucidated. This study aimed to identify the tumor suppressor role of tRF-5026a (tRF-18-79MP9P04) in gastric cancer.

**Methods:**

Quantitative reverse transcription-polymerase chain reaction (qRT-PCR) was first used to detect tRF-5026a expression levels in gastric cancer tissues and patient plasma. Next, the relationship between tRF-5026a levels and clinicopathological features in gastric cancer patients was assessed. Cell lines with varying tRF-5026a levels were assessed by measuring tRF-5026a using qRT-PCR. After transfecting cell lines with a tRF-5026a mimic or inhibitor, cell proliferation, colony formation, migration, apoptosis, and cell cycle were evaluated. The expression levels of related proteins in the PTEN/PI3K/AKT pathway were also analyzed by Western blotting. Finally, the effect of tRF-5026a on tumor growth was tested using subcutaneous tumor models in nude mice.

**Results:**

tRF-5026a was downregulated in gastric cancer patient tissues and plasma samples. tRF-5026a levels were closely related to tumor size, had a certain diagnostic value, and could be used to predict overall survival. tRF-5026a was also downregulated in gastric cancer cell lines. tRF-5026a inhibited the proliferation, migration, and cell cycle progression of gastric cancer cells by regulating the PTEN/PI3K/AKT signaling pathway. Animal experiments showed that upregulation of tRF-5026a effectively inhibited tumor growth.

**Conclusions:**

tRF-5026a (tRF-18-79MP9P04) is a promising biomarker for gastric cancer diagnostics and has tumor suppressor effects mediated through the PTEN/PI3K/AKT signaling pathway.

**Supplementary Information:**

The online version contains supplementary material available at 10.1186/s13287-021-02497-1.

## Introduction

Gastric cancer is one of the most common digestive tract tumors worldwide [1]. Early-stage gastric cancer patients do not typically experience discomfort or any symptoms. Consequently, most gastric cancer patients are diagnosed with middle- and late-stage disease, resulting in a 5-year postoperative survival rate of as low as 25%. However, the 5-year postoperative survival rate for early-stage gastric cancer is 90–95% [2]. Therefore, it is vitally important to study the molecular mechanisms underlying gastric cancer occurrence to help identify biomarkers for the early detection of gastric cancer.

Noncoding RNAs (ncRNAs) are a class of RNAs that do not encode proteins. ncRNA mutations or abnormal expression are closely related to the development of many diseases. MicroRNAs (miRNAs), long noncoding RNAs (lncRNAs), and circular RNAs (circRNAs) have been demonstrated to play a role in the occurrence, development, and prognosis of gastric cancer [3–7].

Recently, tRNA-derived small RNAs (tsRNAs), which were once mistaken for random tRNA degradation products, have been found to have important biological functions [8]. These small ncRNAs are produced by specific cleavage of precursor tRNAs or mature tRNAs at different sites [8–10]. These tsRNAs act as signaling molecules in stress responses and regulate gene expression [11, 12]. Therefore, there is potential for the broad application of tsRNAs in disease diagnosis and treatment. tsRNAs mainly include tRNA-derived fragments (tRFs) and tRNA halves (tiRNAs) [13–16]. tRFs are approximately 14–31 nucleotides (nt) in length and can be further classified based on their source: tRF-1, tRF-2, tRF-3, tRF-5, and i-tRF [13]. tiRNAs can also be divided into two subclasses: 5′ tiRNA and 3′ tiRNA [13].

High-throughput sequencing and database screening have revealed disease-associated tsRNAs [17–19]. In this study, we screened tRF-5026a through tRFdb (http://genome.bioch.virginia.edu/trfdb/) [14] and MINTbase (ID: tRF-18-79MP9P04; https://cm.jefferson.edu/MINTbase/) [17], which indicated that tRF-5026a (tRF-18-79MP9P04) is a gastric cancer-associated tRF. However, its diagnostic value and biological roles in gastric cancer are unclear. tRF-5026a, belonging to the tRF-5 subgroup, comes from mature tRNA^Val−AAC^ and tRNA^Val−CAC^. We found that tRF-5026a had value in the diagnosis of gastric cancer. Furthermore, by upregulating and downregulating the expression of tRF-5026a in gastric cancer cells, we found that tRF-506a regulated the growth of gastric cancer cells through the PTEN/PI3K/AKT signaling pathway. These results helped to increase our understanding of gastric cancer and suggested that tRF-5026a may be useful as a biomarker and therapeutic target of gastric cancer.

## Materials and methods

### Tissue and plasma samples

In this study, 86 pairs of gastric cancer tissues and their corresponding adjacent nontumor tissues were collected at the Affiliated People’s Hospital of Ningbo University, China. Nontumor tissues located 5 cm away from the edge of cancerous tissue were visually confirmed to have no obvious tumor cells. Tissue specimens were immediately preserved in RNAfixer Reagent (Bioteke, Beijing, China) after removal from the patient and kept at – 80 °C until further use. In addition, fasting plasma samples were collected from 37 gastric cancer patients 1 day before and 7 days after surgery. Fresh normal plasma samples were collected from 37 healthy age- and sex-matched normal donors at Ningbo No. 1 Hospital. Ethylenediaminetetraacetic acid (EDTA) was used as the anticoagulant in the tubes used for blood collection.

All tissue samples included in this study were pathologically diagnosed. All clinical data were collected by experienced physicians. The researchers were blinded to the clinical data when operating.

### Cell culture and transfection

The normal gastric mucosal epithelial cell line GES-1 was purchased from the Chinese Academy of Medical Sciences Cancer Hospital (Beijing, China). The gastric cancer cell lines AGS, MGC-803, HGC-27, BGC-823, and SGC-7901 were purchased from the Shanghai Institute of Life Sciences, Chinese Academy of Sciences (Shanghai, China). AGS cells were cultured in Dulbecco’s modified Eagle’s medium (DMEM) with high glucose (HyClone, Logan, UT, USA) supplemented with 1% penicillin/streptomycin (Gibco, Grand Island, NY, USA) and 10% fetal bovine serum (FBS) (PAN-Biotech, Aidenbach, Germany) at 5% CO_2_ and 37 °C. The gastric cancer cell lines MGC-803, HGC-27, BGC-823, and SGC-7901 and the normal gastric mucosal epithelial cell line GES-1 were cultured in Roswell Park Memorial Institute (RPMI) 1640 medium (HyClone, Logan, UT, USA) supplemented with 10% FBS and 1% penicillin/streptomycin at 5% CO_2_ and 37 °C.

Cells were seeded in cell culture plates or culture flasks. When the cells reached 40–60% confluency, the cells were transfected with 0.5 μM tRF-5026a mimic or inhibitor with Lipofectamine 2000 transfection reagent (Life Technologies, Carlsbad, CA, USA). The sequences of the tRF-5026a mimic and mimic negative control used were 5′-GUUUCCGUAGUGUAGUGG-3′ and 5′-UUGUACUACACAAAAGUACUG-3′, respectively. The sequences of the tRF-5026a inhibitor and inhibitor negative control were 5′-CCACUACACUACGGAAAC-3′ and 5′-CAGUACUUUUGUGUAGUACAA-3′, respectively. The oligos were designed and synthesized by GenePharma Co., Ltd. (Shanghai, China).

### Total RNA extraction

Total RNA in tissues and cells was extracted with TRIzol reagent, while RNA was extracted from plasma with TRIzol LS reagent (Invitrogen, Karlsruhe, Germany). The RNA quality was then measured using a SmartSpec Plus Ultra-Micro Spectrophotometer (Bio-Rad, Hercules, CA, USA). The RNA purity was assessed based on the A260/A280 values [19], where values of 1.8–2.1 were considered acceptable. RNA was stored at − 80 °C until further use.

### RNA pretreatment and reverse transcription

The length of tRF-5026a is 18 nt and is therefore too short to detect with quantitative reverse transcription-polymerase chain reaction (qRT-PCR). Adaptors were utilized to mitigate this problem. However, RNA modifications that are typically observed with tRFs and tiRNAs, such as 3′-aminoacyl, methylation, and 2′,3′-cyclic phosphate, were of concern [19]. These modifications can block the end binding of adaptors to the RNA terminus, where internal methylation can hinder cDNA synthesis during reverse transcription (RT). The rtStar™ tRF and tiRNA Pretreatment Kit (Arraystar, Rockville, MD, USA) was used to remove these modifications. The 3′ adaptor contained a universal RT primer (5′-AGATCGGACGCGG-3′). The 5′ adaptor and 3′ adaptor sequences used were 5′-TCGGCCGACGATC-3′ and 5′-CCGCGTCCGATCT-3′, respectively. The manufacturer’s protocol was followed for RNA pretreatment (demodification) [19]. The rtStar™ First-Strand cDNA Synthesis Kit (Arraystar) was then used to synthesize cDNA for the detection of tRFs with qRT-PCR.

### PCR analysis

Upon adding cDNA, the PCR mix was assembled with GoTaq qPCR Master Mix (Promega, Madison, WI, USA) according to the manufacturer’s protocol. PCR was performed on an Mx3005P Real-Time PCR machine (Stratagene, Palo Alto, CA, USA). Small nuclear RNA RNU6-2 was used as an external reference control for tRF-5026a. The Δ*C*_q_ method was used to analyze the expression levels, where a lower Δ*C*_q_ value indicates a higher expression. The relative expression was calculated using the 2^−ΔΔ*C*q^ method. Data are expressed as the mean ± standard deviation (SD) of experiments conducted in triplicate. The primer sequences for qRT-PCR are shown in Supplementary Table S1.

To verify the accuracy of qRT-PCR, the qRT-PCR products were subjected to agarose gel electrophoresis and sequencing. The tRF-5026a qRT-PCR product was first purified using the UNIQ-10 PCR Product Purification Kit (Thermo Fisher Scientific, Waltham, MA, USA) and cloned into the pUCm-T vector (Thermo Fisher Scientific) according to the manufacturer’s instructions. Sequencing was performed by Thermo Fisher Scientific. In addition, we measured the tRF-5026a levels with Northern blotting by designing a specific complementary probe sequence (5′-CCACTACACTACGGAAAC-3′).

### Cell proliferation assay

Cell proliferation was assayed with a Cell Counting Kit 8 (CCK-8) (Dojindo Molecular Technologies, Kumamoto, Japan). Specifically, 24 h after transfection with the tRF-5026a mimic or inhibitor, 5 × 10^3^ cells in 100 μL were seeded per well of a 96-well plate. Each treatment was performed with six replicates. After incubating the cells in a CO_2_ incubator for 24 h, a total of 10 μL of CCK-8 solution was added to each well, and the cells were incubated for an additional 3 h. Finally, the absorbance was measured at 450 nm with a microplate reader (SpectraMax M5, Molecular Devices, CA, USA).

### Colony formation assay

tRF-5026a mimic- or inhibitor-transfected cells were added to a 6-well plate (500 cells/well) 24 h post transfection and cultured in a CO_2_ incubator. Each condition was performed in triplicate. After culturing the cells for 15 days, the cells were gently rinsed with phosphate-buffered saline (PBS) once or twice and then fixed with 1 mL/well of a 4% paraformaldehyde solution for 30 min. The cells were stained with 1 mL of 0.1% crystal violet dye for 30 min, and the plate was rinsed gently with tap water several times. After drying, a picture was taken and used for analysis.

### Cell migration assay

A Transwell assay was used to measure cell migration. Transfected cells were first incubated in a CO_2_ incubator for 24 h and then resuspended in Opti-MEM I Reduced-Serum Medium (Gibco). A total of 8 × 10^4^ cells (200 μL) were added to the upper chamber of a Transwell insert (Costar, Corning, NY, USA), and 500 μL of RPMI 1640 medium containing 10% FBS was added to the lower chamber. After the cells were cultured at 37 °C in a CO_2_ incubator for 24 h, the cells were fixed and stained with paraformaldehyde fixation and crystal violet dye. The migrating cells were then counted.

### Cell cycle detection

The cells were first starved for one day in serum-free medium to synchronize the cell cycles. After transfection, the cells were collected in a flow tube. One milliliter of DNA staining solution and 10 μL of permeabilization solution (Multi Sciences) were added. After vortexing the tubes for 5–10 s, the cells were incubated for 30 min in the dark at room temperature. Finally, the stained cells were run on a FACSCalibur Flow Cytometer (BD Biosciences). The data were analyzed with Modifit software (BD Biosciences).

### Western blot analysis

The cells were first lysed with radioimmunoprecipitation assay lysis buffer (Solarbio, Beijing, China). The protein levels were then quantified using a Bradford assay kit (Beyotime, Haimen, China). After the protein samples were separated by 12% SDS-polyacrylamide gel electrophoresis, the proteins were transferred to a polyvinylidene fluoride (PVDF) membrane (Millipore, Billerica, MA, USA). The membranes were blocked and incubated with primary antibodies, followed by washing with Tris-buffered saline and Tween 20 (TBST). Secondary antibodies were then incubated with the membranes followed by washing with TBST. Finally, the proteins were detected using WesternBright ECL HRP (Advansta, Menlo Park, CA, USA) and the signals were detected with a Clinx GenoSens 1600 integrated gel imaging analysis system (Clinx, Shanghai, China). The primary antibodies that were directed against phosphates and tensin homolog deleted on chromosome ten gene (PTEN), phosphatidylinositide 3-kinase (PI3K), and protein kinase B (AKT), as well as the goat anti-mouse IgG and goat anti-rabbit IgG secondary antibodies, were all purchased from Cell Signaling Technology Company (Danvers, MA, USA).

### Subcutaneous tumor model

Male 3-week-old BALB/c nude mice purchased from the Shanghai Siliake Laboratory Animal Center (Siliake, Shanghai, China) were housed in a specific pathogen-free environment in the Experimental Animal Center of Ningbo University, China. After consulting the literature, we found that gastric cancer SGC-7901 and MGC-803 cells have good tumorigenic properties [20, 21]. Therefore, the SGC-823 and MGC-803 cells were used for xenograft studies. The mice were split into the following groups: mimic negative control, low concentration mimic (0.05 μM), and high concentration mimic (0.1 μM). Each group contained six mice each. A total of 5 × 10^6^ cells in 150 μL were injected subcutaneously. Treatments were given every other week. After tumor formation, the tumor volumes were measured every other day, and the mice were sacrificed 1 month later. After the tumors were removed, their sizes and masses were measured. The major organs were also assessed macroscopically for changes.

### Statistical analysis

The data are expressed as the mean ± SD and were analyzed using Statistical Program for Social Sciences (SPSS) 20.0 (IBM, Chicago, IL, USA) software. Differences between groups were evaluated using a two-sided Student’s *t*-test. *P* < 0.05 was considered meaningful.

## Results

### Low expression of tRF-5026a is observed in gastric cancer tissues and cells

**Fig. 1:**
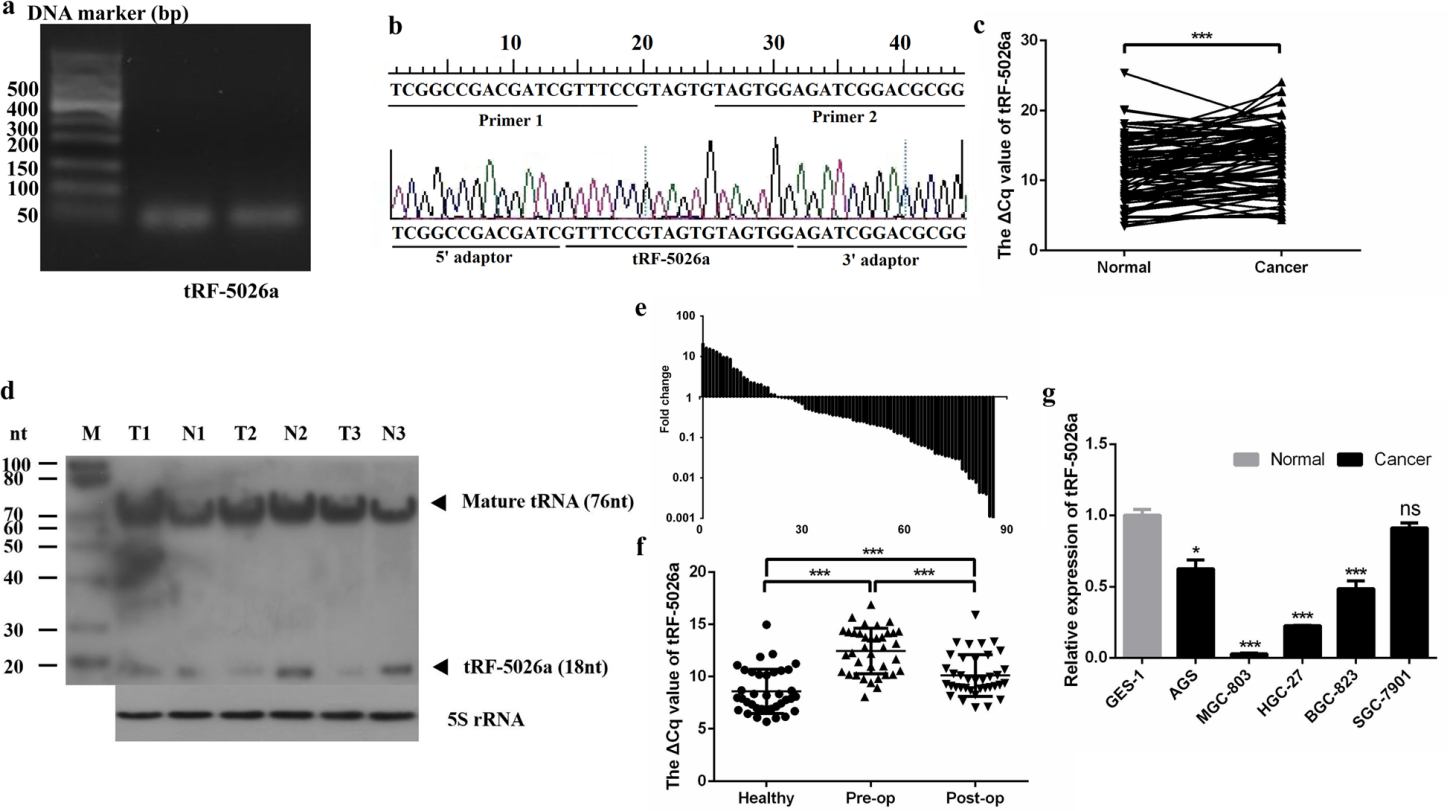
Verification of tRF-5026a expression in tissues, plasma samples, and cells. **a** Electrophoretogram of tRF-5026a qRT-PCR products from two representative gastric cancer tissues. **b** T-A clone sequencing results of tRF-5026a qRT-PCR products with the original tRF-5026a sequence shown, the sequence of the two adaptors, and the crossing joint primer sequences. **c** Expression levels of tRF-5026a in gastric cancer tissues (*n* = 86). A larger Δ*C*_q_ value indicates lower expression. **d** tRF-5026a (18 nt) and its mature tRNA (76 nt) were detected by Northern blotting. Three representative paired gastric cancer tissues (T) and adjacent noncancerous tissues (N) are shown. The 5S rRNA was used as a loading control. **e** The expression level of tRF-5026a was significantly downregulated in 79.07% (68/86) of gastric cancer tissues compared with normal tissues. **f** tRF-5026a levels in plasma samples from gastric cancer patients and healthy controls. A larger Δ*C*_q_ value indicates a lower expression. *n* = 37. **g** tRF-5026a expression levels in cell lines. Low expression levels of tRF-5026a in gastric cancer cell lines were found compared with the normal gastric mucosal epithelial cell line GES-1. ^*^P < 0.05, ^**^P < 0.01, ^***^P < 0.001, ns, no significance

To detect tRF-5026a levels by qRT-PCR, specific amplification primers for tRF-5026a were designed that spanned both the tRF-5026a and adaptor sequences. Agarose gel electrophoresis showed that the qRT-PCR product length was consistent with its theoretical length (44 bp) (Fig. 1a). To further confirm the qRT-PCR product was correct, T-A cloning and sequencing were performed. The alignment results (Fig. 1b) were consistent with the base sequence of tRF-5026a obtained from the MINTbase database (https://cm.jefferson.edu/MINTbase/) and the tRFdb database (http://genome.bioch.virginia.edu/trfdb/search.php), indicating that the primers for tRF-5026a were capable of specifically amplifying tRF-5026a.

To understand the expression of tRF-5026a in gastric cancer, 86 pairs of gastric cancer and adjacent tissues were collected. tRF-5026a was expressed at low levels in gastric cancer tissues, as assessed by qRT-PCR (Fig. 1c). We also measured the expression of tRF-5026a in gastric cancer and paracancerous tissues using Northern blotting. Northern blotting confirmed that tRF-5026a was indeed expressed at low levels in gastric cancer tissues and that the length of tRF-5026a (18 nt) and its mature tRNA (76 nt) were consistent with the theoretical length (Fig. 1d). From the qRT-PCR data, we observed that 79.1% (68/86) of the samples expressed low amounts of tRF-5026a (Fig. 1e).

Plasma is one of the most commonly collected sample types in the clinic. We therefore measured plasma tRF-5026a levels in gastric cancer patients and healthy controls. Healthy people had higher plasma tRF-5026a levels than gastric cancer patients both one day prior to and 7 days postoperatively (Fig. 1f). In addition, tRF-5026a plasma levels of postoperative patients were higher than those of preoperative patients (Fig. 1f); that is, after the tumor was removed by surgery, the plasma tRF-5026a level tended to increase to the level of healthy people.

To further investigate tRF-5026a expression in gastric cancer, we explored the expression levels of tRF-5026a at the cellular level. tRF-5026a was expressed at lower levels in gastric cancer cells (AGS, MGC-803, HGC-27, BGC-823, and SGC-7901) than in normal gastric mucosal epithelial cells (GES-1), as assessed by qRT-PCR (Fig. 1g). These cell line data were consistent with the data from the tissue and plasma samples (Fig. 1e, f).

### Diagnostic value of tRF-5026a in gastric cancer

Given that the levels of tRF-5026a were different in malignant tissues, plasma samples, and cell lines compared to normal conditions (Fig. 1), tRF-5026a therefore has the potential to serve as a biomarker of gastric cancer. To assess this potential, we first analyzed the area under the receiver operating characteristic (ROC) curve (AUC) of tRF-5026a and then measured the relationship between tRF-5026a expression levels in gastric cancer tissues and the clinicopathological features of gastric cancer patients. Using the postoperative follow-up data of gastric cancer patients, survival curves were drawn to evaluate the outcomes of the gastric cancer patients. Finally, independent predictors of gastric cancer prognosis were identified by single factor and multivariate Cox regression analysis.

**Fig. 2:**
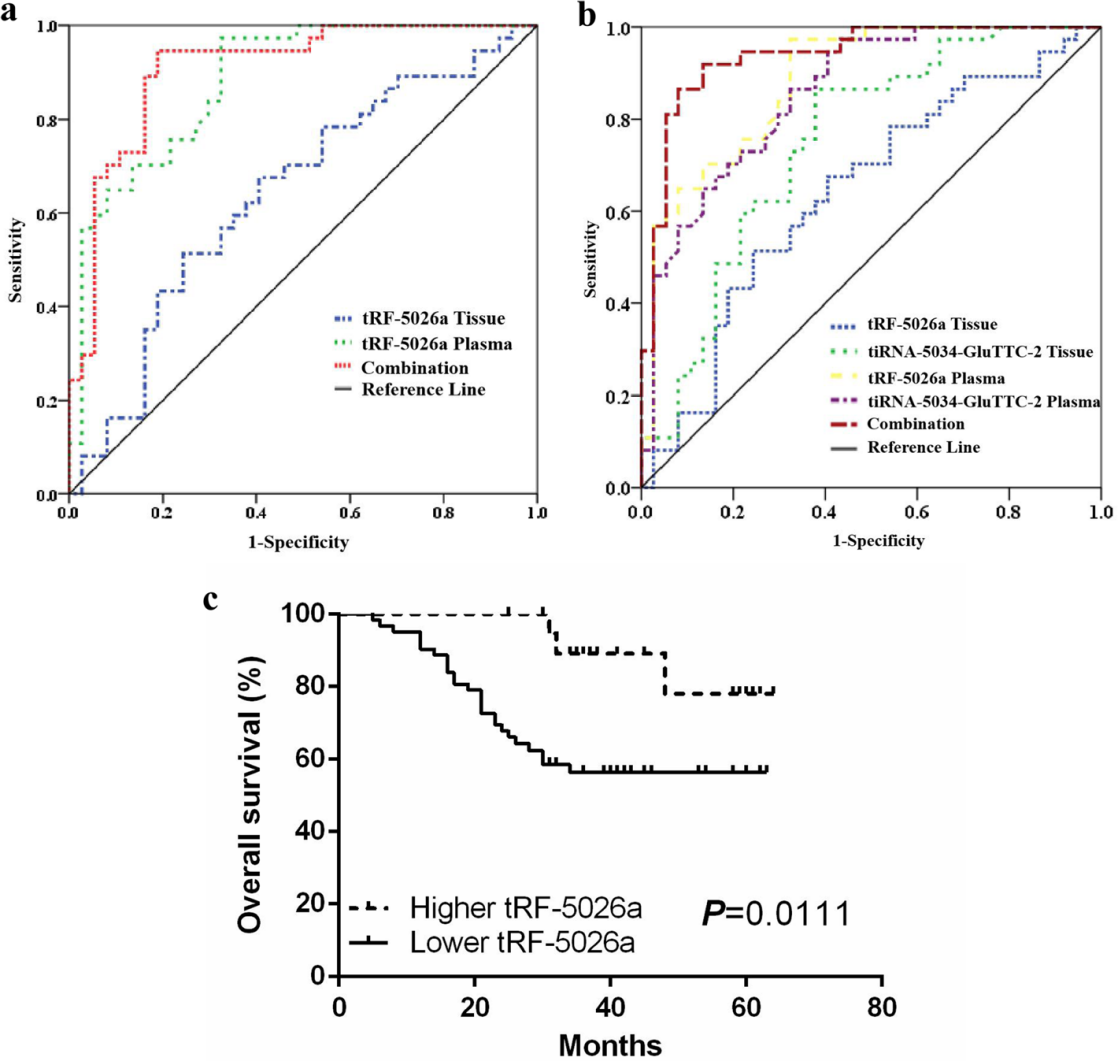
tRF-5026a may be a potential diagnostic biomarker. **a** Diagnostic value analysis of tRF-5026a in gastric cancer. **b** Combined diagnostic assessment of tRF-5026a and tiRNA-5034-GluTTC-2 in tissues and plasma samples. **c** Prognostic significance of tRF-5026a in gastric cancer patients. Kaplan-Meier analysis of overall survival (OS) based on tRF-5026a expression levels in tissues

For tissue expression of tRF-5026a, the AUC was 0.631, and the sensitivity and specificity were 0.512 and 0.721, respectively, for a cutoff value of 14.03 (Fig. 2a). For plasma expression of tRF-5026a, the AUC was 0.883, and the sensitivity and specificity were 0.973 and 0.676, respectively, for a cutoff value of 8.81 (Fig. 2a). The diagnostic efficiency of tRF-5026a as a biomarker was significantly improved when using a combination of tissue and plasma expression data. The combined AUC was 0.908, with a sensitivity and specificity of 0.946 and 0.811, respectively (Fig. 2a).

The combined use of several biomarkers is a useful method to improve their diagnostic value [22]. We previously found that tiRNA-5034-GluTTC-2 could be a potential biomarker of gastric cancer [19]. We combined the tRF-5026a and tiRNA-5034-GluTTC-2 data to determine whether the diagnostic value increased. When we combined the tissue and plasma levels of both tRF-5026a and tiRNA-5034-GluTTC-2, the AUC increased to 0.938, with a sensitivity of 0.919 and a specificity of 0.865 (Fig. 2b). The AUCs of the tissue and plasma tRF-5026a and tiRNA-5034-GluTTC-2 were higher than when they were used separately (Supplementary Table S2).

In addition, after analyzing the clinicopathological features of gastric cancer patients, we found that tRF-5026a expression levels were associated with tumor serum marker carbohydrate antigen 19-9 (CA19-9) levels (*P* = 0.026) and tumor size (*P* = 0.001) (Supplementary Table S3). A survival curve analysis found that tRF-5026a expression levels in tissues from gastric cancer patients correlated with overall survival (OS), where the OS of the low tRF-5026a expression group was shorter than that of the high expression group (Fig. 2c). Univariate and multivariate analyses showed that tRF-5026a was associated with TNM stage and lymph node metastasis and was a good independent prognostic biomarker for gastric cancer (Supplementary Table S4).

### Effects of tRF-5026a on cell proliferation

To study the role of tRF-5026a in gastric cancer, we used a tRF-5026a mimic and inhibitor to modulate the expression levels in gastric cancer cells. The tRF-5026a levels were upregulated by the tRF-5026a mimic in a normal gastric mucosal epithelial cell line (GES-1) and gastric cancer cell lines (AGS, MGC-803, HGC-27, BGC-823, and SGC-7901), as measured by qRT-PCR (Supplementary Fig. S1a). We also successfully downregulated tRF-5026a expression in the normal gastric mucosal epithelial cell line GES-1 and the gastric cancer cell lines AGS, HGC-27, BGC-823, and SGC-7901 (Supplementary Fig. S1b).

The CCK-8 assay revealed that in the normal gastric mucosal epithelial cell line GES-1 (Supplementary Fig. S2a, b) and the gastric cancer cells AGS (Supplementary Fig. S2c, d), BGC-823 (Supplementary Fig. S2e, f), and SGC-7901 (Supplementary Fig. S2 g, h), increasing tRF-5026a levels inhibited proliferation, while decreasing tRF-5026a promoted proliferation.

**Fig. 3:**
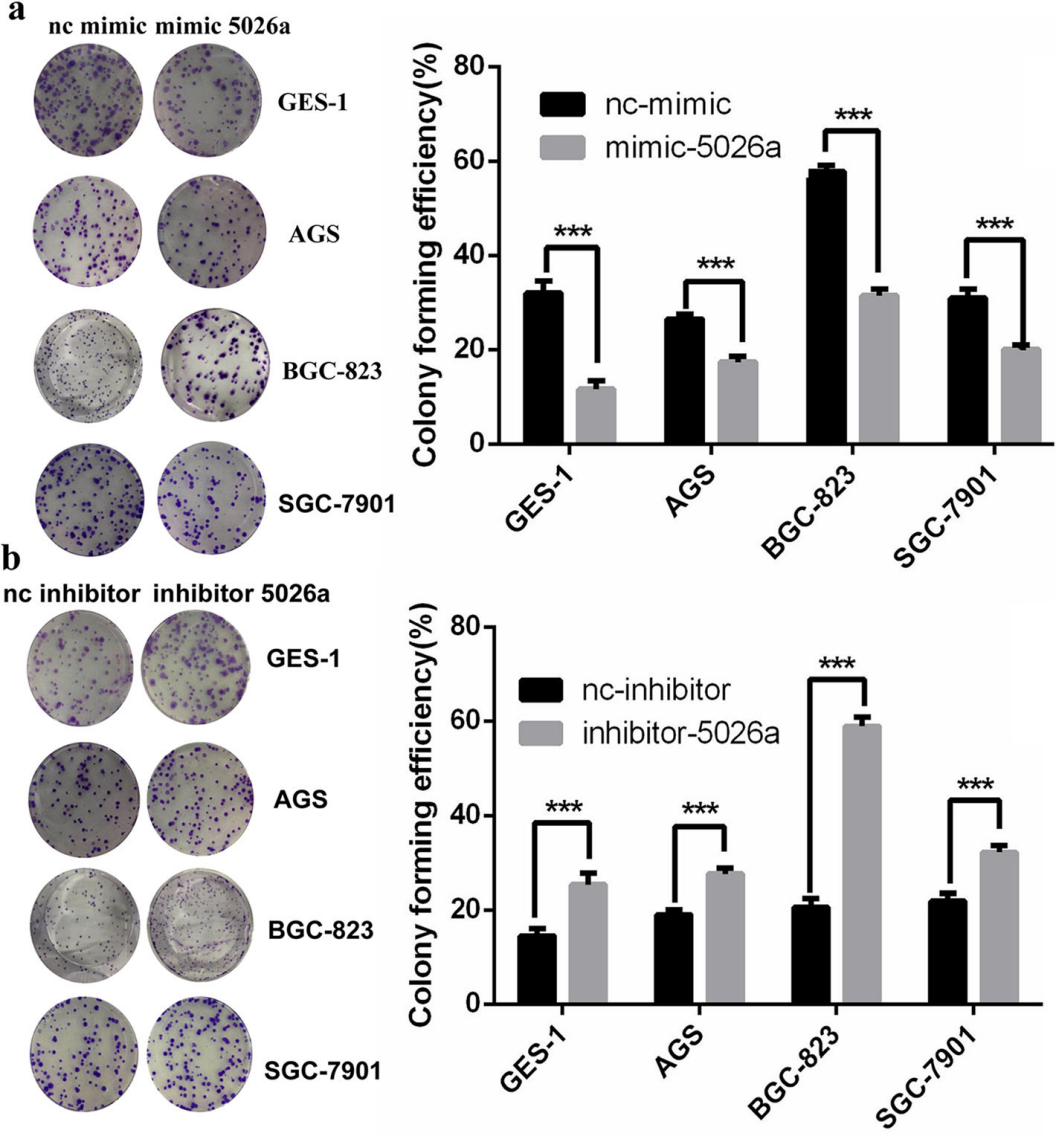
Effects of tRF-5026a on colony formation. **a** Colony formation assay of tRF-5026a mimic with the normal gastric mucosal epithelial cell line (GES-1) and gastric cancer cell lines (AGS, BGC-823, SGC-7901). **b** Colony formation assay of the tRF-5026a inhibitor with the normal gastric mucosal epithelial cell line (GES-1) and gastric cancer cell lines (AGS, BGC-823, SGC-7901). Left, representative images. Right, data are presented as the mean ± SD; nc, negative control. *n* = 3, ^**^P < 0.01, ^***^P < 0.001

We further verified the suppressive effects of tRF-5026a on the proliferative capacity by using a colony formation assay. In the normal gastric mucosal epithelial cell line GES-1 and the gastric cancer cells AGS, BGC-823, and SGC-7901, the tRF-5026a mimic decreased colony formation, while the inhibitor increased colony formation (Fig. 3).

### Effects of tRF-5026a on cell migration

The effects of tRF-5026a levels on cell migration were analyzed using a Transwell assay. The migratory capabilities of the normal gastric mucosal epithelial cell line GES-1 and the gastric cancer cell lines AGS, BGC-823, and SGC-7901 were decreased with the tRF-5026a mimic and increased by the inhibitor (Supplementary Fig. S3).

### Effects of tRF-5026a on the cell cycle

**Fig. 4:**
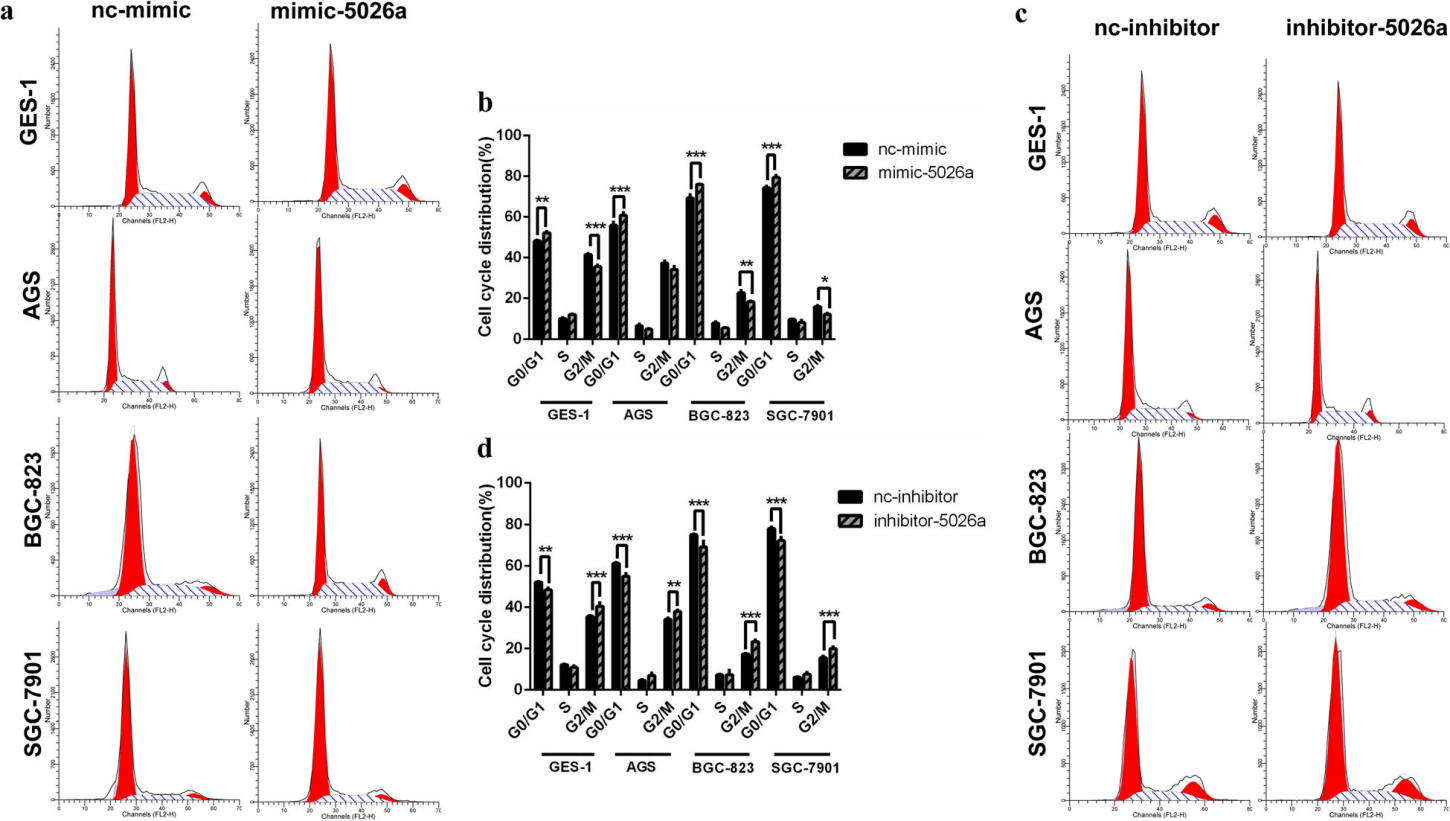
Effects of tRF-5026a on cell cycle distribution measured by flow cytometry. **a**–**d** Cell cycle distribution in the normal gastric mucosal epithelial cell line (GES-1) and the gastric cancer cell lines (AGS, BGC-823, SGC-7901) following increases and decreases of tRF-5026a levels with the tRF-5026a mimic (**a**, **b**) and inhibitor (**c**, **d**), respectively. **a**, **c** Representative plots. **b**, **d** Data are presented as the mean ± SD, *n* = 3; nc, negative control, ^*^P < 0.05, ^**^P < 0.01, ^***^P < 0.001

To explain the effects of tRF-5026a on proliferation in gastric cancer (Supplementary Fig. S2, Fig. 3), we next measured the effects of tRF-5026a on the cell cycle. Increasing tRF-5026a levels resulted in a G_0_/G_1_ block in the normal gastric mucosal epithelial cell line GES-1 and the gastric cancer cell lines AGS, BGC-823, and SGC-7901, as measured by flow cytometry (Fig. 4). Interestingly, decreasing tRF-5026a levels caused a block at G_2_/M in these cell lines. These findings indicated that tRF-5026a regulated cell cycle progression.

### Effects of tRF-5026a on the expression of signal transduction-related proteins

To further investigate the effects of tRF-5026a on proliferation, migration, and cell cycle (Figs. 3 and 4), we next examined whether tRF-5026a was involved in the PTEN/PI3K/AKT signaling pathway because this pathway is a fundamental player in biological activities such as cell proliferation, migration, and the cell cycle [23, 24]. PI3K and AKT are positive regulators, while PTEN is a negative regulator of this pathway.

**Fig. 5:**
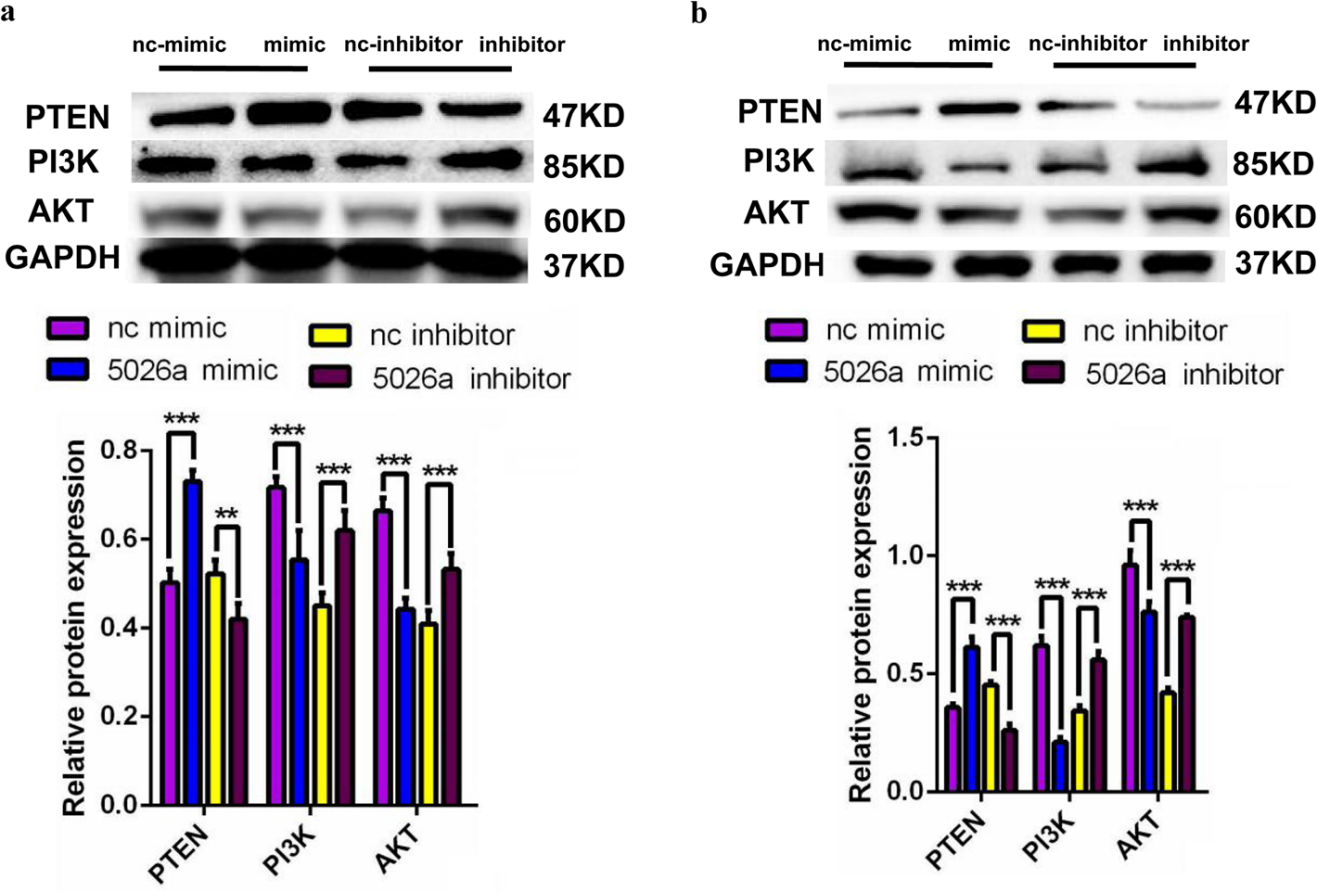
Effects of tRF-5026a on the expression of PTEN/PI3K/AKT signaling pathway-associated proteins in BGC-823 cells (**a**) and SGC-7901 cells (**b**). The top panel shows a Western blot image, and the bottom panel shows quantification of the signal. Data are presented as the mean ± SD, *n* = 3; nc, negative control; * *P* < 0.05, ** *P* < 0.01, *** *P* < 0.001

We selected the poorly differentiated gastric cancer cell line BGC-823 and the moderately differentiated gastric cancer cell line SGC-7901 as representative gastric cancer cell lines to study the effects of tRF-5026a on the expression of PTEN, PI3K, and AKT. We found that increasing tRF-5026a levels in gastric cancer cells resulted in a decrease in PI3K and AKT levels, with an increase in PTEN levels (Fig. 5). Conversely, decreasing tRF-5026a levels resulted in an increase in PI3K and AKT levels and a decrease in PTEN levels (Fig. 5).

### Effects of tRF-5026a on the growth of transplanted tumors in animal models

**Fig. 6:**
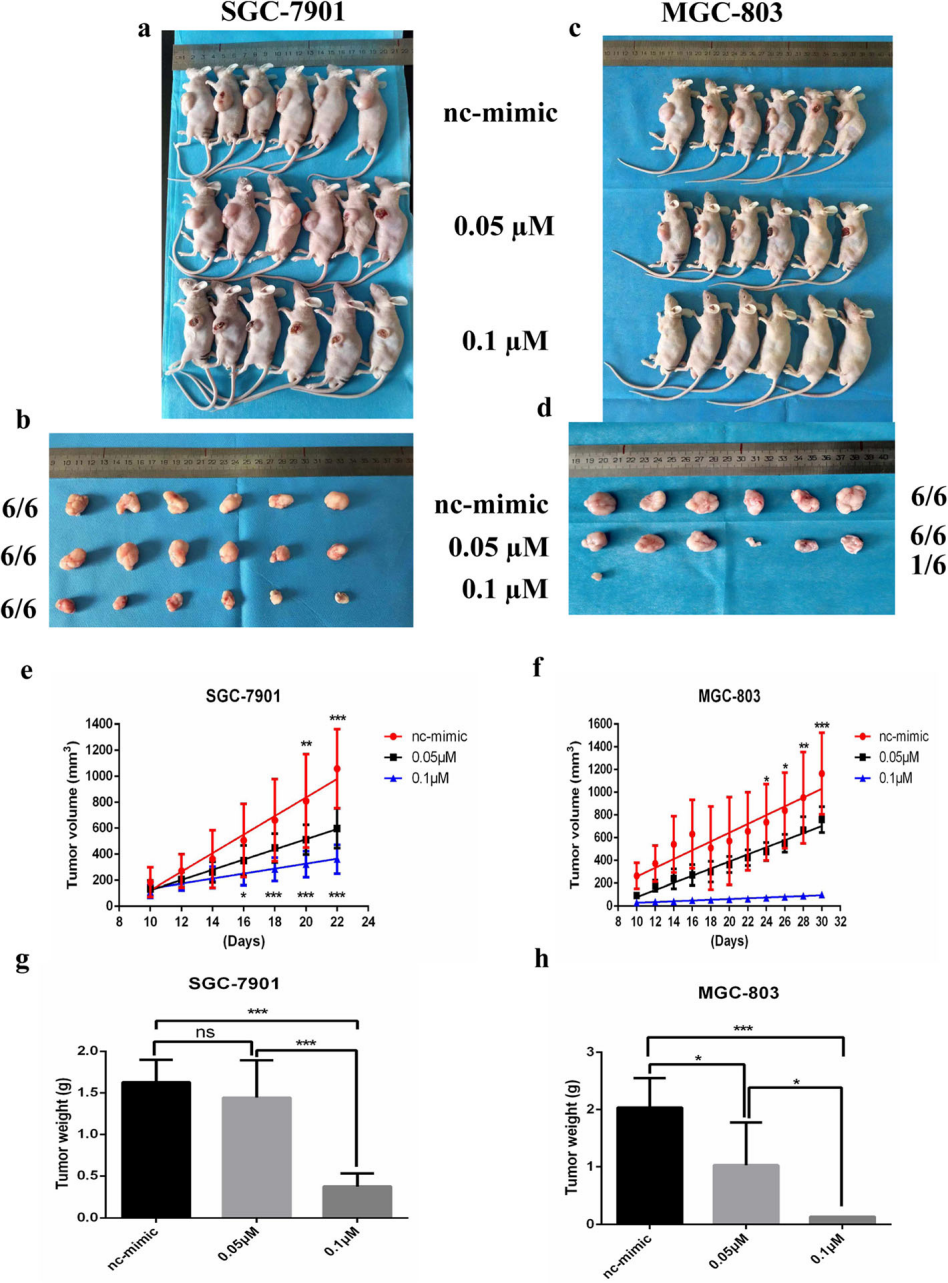
Effects of tRF-5026a on transplanted tumor growth of the gastric cancer cell lines SGC-7901 (**a**, **b**) and MGC-803 (**c**, **d**). Tumor volume changes of SGC-7901 cell (**e**) and MGC-803 cell (**f**) transplants with various doses of the tRF-5026a mimic. Tumor weight changes in SGC-7901 cell (**g**) and MGC-803 cell (**h**) transplants with various concentrations of the tRF-5026a mimic. nc, negative control. ^*^P < 0.05, ^**^P < 0.01, ^***^P < 0.001; ns, no significance

To further elucidate the role of tRF-5026a in gastric cancer, we performed a subcutaneous tumor formation experiment in nude mice. Increasing tRF-5026a levels in the gastric cancer cell lines SGC-7901 (Fig. 6a, b) and MGC-803 (Fig. 6c, d) significantly inhibited the tumor growth rate as measured by the tumor volume (Fig. 6e, g) and tumor weight changes (Fig. 6f, h) in a dose-dependent manner compared with the control group. Moreover, tumor growth in the gastric cancer cell line MGC-803 was completely prevented in 5/6 mice in the high concentration tRF-5026a mimetic group (Fig. 6c, d). The possible reasons are as follows: (1) The baseline level of tRF-5026a in MGC-803 cells was lower than that in SGC-7901 cells (Fig. 1g); and (2) the upregulation effects of the tRF-5026a mimic on the level of tRF-5026a between MGC-803 cells and SGC-7901 cells were not significantly different (Fig. S1). These results indicate that MGC-803 cells are more sensitive to the tRF-5026a mimic than SGC-7901 cells.

Although there were no significant differences between the baseline levels of tRF-5026a in GES-1 and SGC-7901 cells (Fig. 1g), we did not use the GES-1 cells in the animal experiment because it is a normal gastric mucosal epithelial cell line and does not form xenografts.

## Discussion

The main role of tRNAs is to carry amino acids to the ribosome, thereby facilitating the synthesis of the corresponding protein under the guidance of the mRNA. In recent years, it has been found that many tsRNAs (tRFs and tiRNAs) produced under specific conditions are in fact not random tRNA degradation products [25]. Moreover, many studies have recently shown that tRFs and tiRNAs can be found in a variety of cancers [26–29]. tRFs and tiRNAs can affect the development of cancer by regulating transcription, altering mRNA stability, inhibiting translation, and regulating ribosome biogenesis [15]. tRFs and tiRNAs can also affect cancer development by regulating cell proliferation, metastasis, apoptosis, and the cell cycle [30, 31]. These tsRNAs can influence the expression levels of endogenous target genes [25, 32, 33]. Some tRFs and tiRNAs also form complexes with Ago and Piwi, indicating that these tRFs and tiRNAs can function as miRNAs or piRNAs [26]. tRFs and tiRNAs also serve as regulators of gene expression and the stress response [34].

The rich modifications observed on tRFs and tiRNAs [8, 9] increase their stability in tissues, plasma, and cells compared with other ncRNAs (e.g., lncRNAs, circRNAs, and miRNAs) [35–37]. In addition, tRFs and tiRNAs are highly enriched in plasma samples and other bodily fluids [38, 39]. Pretreatment of total RNAs and the addition of an adaptor prior to qRT-PCR results in higher yields and lower dimer production rates when trying to measure the levels of these tsRNAs [40]. In addition, tRFs and tiRNAs are abnormally expressed in cancer and can be detected efficiently. tRFs and tiRNAs are therefore prospective new biomarkers for the noninvasive diagnosis of gastric cancer.

Studies have found that tsRNAs are differently expressed in stem cells who are in different differentiation states and affect their genes’ transcription and translation [41]. Huang et al. found that tRF/miR-1280 can promote cancer stem cells (CSCs) in the progression of colorectal cancer [28]. Many tsRNAs were detected in embryonic stem cells (ESCs) and induced pluripotent stem cells [42]. Guzzi et al. found that in mammalian stem cells, post-transcriptional RNA modification affected the biogenesis and function of tRFs [43]. PTEN/PI3K/Akt pathway is related to CSCs in various cancers [44–46]. Dubrovska et al. found that the PTEN/PI3K/Akt pathway was closely related to prostate CSCs and PI3K might be an effective therapeutic target of prostate cancer [45]. In non-small cell lung cancer (NSCLC), PI3K/AKT pathway plays a great role in the enrichment of CSCs, thereby promoting the occurrence and development of NSCLC [46]. And some small RNAs such as miR-873 inhibits the proliferation and differentiation of pancreatic CSCs mediated through PI3K/AKT signaling pathway [47]. Here, we found that tRF-5026a inhibited the occurrence and development of gastric cancer through PI3K/AKT signaling pathway.

This study mainly aimed to screen for and identify gastric cancer-related tRFs and to explore their biological functions. tRFs have the potential to play a major role in gastric cancer diagnostics because tRFs are more stable than traditional ncRNAs, and many ncRNAs have demonstrated potential utility in gastric cancer diagnostics. It remains to be determined how tRFs regulate the occurrence and development of gastric cancer.

In this study, we measured representative gastric cancer-associated tRFs at three levels, the cellular, animal, and clinical levels, and studied their possible biological functions. We found that tRF-5026a was downregulated in gastric cancer tissues, plasma samples and cells (Fig. 1), indicating that tRF-5026a may have a tumor suppressor role and can be used as a potential biomarker for gastric cancer diagnosis (Fig. 2). We found that increasing tRF-5026a levels inhibited cell proliferation and migration and arrested the cell cycle process, whereas silencing tRF-5026a resulted in increased proliferation and migration (Figs. 3 and 4).

We then evaluated whether tRF-5026a could affect the development of gastric cancer by acting on critical signaling pathways. Because the PI3K/AKT signaling pathway is one of the fundamental pathways identified in the development of gastric cancer, we measured the effects of tRF-5026a on the expression of key proteins in this pathway. The PI3K/AKT signaling pathway plays a role in promoting cell proliferation, preventing cell apoptosis, and promoting cell viability [48]. PI3K and AKT are positive regulators of this pathway, while PTEN acts as a negative regulator of the PI3K/AKT signaling pathway. PI3K is dephosphorylated to phosphatidylinositol diphosphate (PIP2) by using phosphatidylinositol triphosphate (PIP3) as a substrate. Because PIP3 is a product of PI3K that mediates the activation of AKT and dephosphorylation to PIP2 is accomplished under the action of PTEN, PTEN therefore inhibits the activity of the PI3K/AKT signaling pathway [49, 50]. We found that upregulating tRF-5026a levels inhibited the PI3K/AKT signaling pathway, while downregulating tRF-5026a promoted activation of the PI3K/AKT pathway (Fig. 5). These effects led to either inhibited cell proliferation and migration, arrested cell cycle progression, or increased proliferation and migration (Figs. 3 and 4). The PI3K/AKT signaling pathway, which is overactive in many cancers, regulates the proliferation, migration, invasion, and cell cycle progression of cancer cells and is closely related to tumor neovascularization, endothelial growth, and replication potential [51]. Our study demonstrated that tRF-5026a inhibited the growth of gastric cancer cells by regulating the PI3K/AKT signaling pathway and it exerted a tumor suppressor effect (Fig. 5). However, one of the limits of this study is that the RNAs and proteins that directly bind to tRF-5026a were not identified, which needs to be addressed in future studies.

In conclusion, tRF-5026a (tRF-18-79MP9P04) is a potential biomarker for the diagnosis of gastric cancer. tRF-5026a (tRF-18-79MP9P04) had tumor suppressive effects in gastric cancer mediated through the PTEN/PI3K/AKT signaling pathway.

## Supplementary Information

**Additional file 1:** **Supplementary Table S1.** Primer sequences for qRT-PCR. **Supplementary Table S2.** Sensitivity, specificity, AUC of tRF-5026a and tiRNA-5034-GluTTC-2 in tissues and plasma samples and their combination. **Supplementary Table S3.** The relationships between the expression levels of tRF-5026a (Δ*C*_q_) in tissues and the clinicopathological factors of patients with gastric cancer. **Supplementary Table S4.** Univariate and multivariate Cox regression analysis of the overall survival of tRF-5026a expression in patients with gastric cancer tissues. **Supplementary Fig. S1.** The use of tRF-5026a mimic and inhibitor to modulate its levels in normal gastric mucosal epithelial cells and gastric cancer cells. **a** The up-regulation effect of tRF-5026a mimic. **b** The down-regulation effect of tRF-5026a inhibitor. nc, negative control; *n*=3, * *P*<0.05, ** *P*<0.01, *** *P*<0.001. **Supplementary Fig. S2.** Effects of tRF-5026a on gastric cancer cell proliferation. **a, b** Grow curve of normal gastric mucosal epithelial cell line (GES-1) following up- and down-regulation by tRF-5026a mimic and inhibitor, respectively. **c-h** Grow curve of gastric cancer cell lines (AGS, BGC-823, SGC-7901) following up- and down-regulation by tRF-5026a mimic and inhibitor, respectively. nc, negative control; *n*=6, ** *P*<0.01, *** *P*<0.001. **Supplementary Fig. S3.** Effects of tRF-5026a on cell migration. **a** Transwell assay of tRF-5026a mimic of the normal gastric mucosal epithelial cell line (GES-1) and gastric cancer cell lines (AGS, BGC-823, SGC-7901). **b** Transwell assay of the tRF-5026a inhibitor of the normal gastric mucosal epithelial cell line (GES-1) and gastric cancer cell lines (AGS, BGC-823, SGC-7901). Left, representative results. Right, data are presented as the mean ± SD, *n*=3, nc, negative control, * *P*<0.05, ** *P*<0.01, *** *P*<0.001.

## Data Availability

The authors declare that the data supporting the findings of this study are available in the article and associated Supplementary Information. Extra data or information are available from the corresponding authors upon reasonable request.

## Abbreviations

AKT: Protein kinase B
AUC: Receiver operating characteristic curve
CA19-9: Carbohydrate antigen 19-9
CCK-8: Cell counting kit 8
circRNAs: Circular RNAs
DMEM: Dulbecco’s modified Eagle’s medium
EDTA: Ethylenediaminetetraacetic acid
FBS: Fetal bovine serum
lncRNAs: Long noncoding RNAs
miRNAs: MicroRNAs
ncRNAs: Noncoding RNAs
nt: Nucleotides
OS: Overall survival
PBS: Phosphate-buffered saline
PI3K: Phosphatidylinositide 3-kinase
PIP2: Phosphatidylinositol diphosphate
PIP3: Phosphatidylinositol triphosphate
PTEN: Tensin homolog deleted on chromosome ten gene
PVDF: Polyvinylidene fluoride
qRT-PCR: Quantitative reverse transcription-polymerase chain reaction
ROC: Receiver operating characteristic
RT: Reverse transcription
SD: Standard deviation
SPF: Specific pathogen-free
TBST: Tris-buffered saline and Tween 20
tiRNAs: tRNA halves
tRFs: tRNA-derived RNA fragments
tsRNAs: tRNA-derived small RNAs
