# Supplementary material for: The tRNA-derived fragment 5026a inhibits the proliferation of gastric cancer cells by regulating the PTEN/PI3K/AKT signaling pathway
Source: Stem Cell Res Ther. 2021 Jul 22;12:418. doi: 10.1186/s13287-021-02497-1 (PMC8296675; doi:10.1186/s13287-021-02497-1)
Supplement: Supplementary file 1 — Additional file 1: Supplementary Table S1. Primer sequences for qRT-PCR. Supplementary Table S2. Sensitivity, specificity, AUC of tRF-5026a and tiRNA-5034-GluTTC-2 in tissues and plasma samples and their combination. Supplementary Table S3. The relationships between the expression levels of tRF-5026a (ΔCq) in tissues and the clinicopathological factors of patients with gastric cancer. Supplementary Table S4. Univariate and multivariate Cox regression analysis of the overall survival of tRF-5026a expression in patients with gastric cancer tissues. Supplementary Fig. S1. The use of tRF-5026a mimic and inhibitor to modulate its levels in normal gastric mucosal epithelial cells and gastric cancer cells. a The up-regulation effect of tRF-5026a mimic. b The down-regulation effect of tRF-5026a inhibitor. nc, negative control; n=3, * P<0.05, ** P<0.01, *** P<0.001. Supplementary Fig. S2. Effects of tRF-5026a on gastric cancer cell proliferation. a, b Grow curve of normal gastric mucosal epithelial cell line (GES-1) following up- and down-regulation by tRF-5026a mimic and inhibitor, respectively. c-h Grow curve of gastric cancer cell lines (AGS, BGC-823, SGC-7901) following up- and down-regulation by tRF-5026a mimic and inhibitor, respectively. nc, negative control; n=6, ** P<0.01, *** P<0.001. Supplementary Fig. S3. Effects of tRF-5026a on cell migration. a Transwell assay of tRF-5026a mimic of the normal gastric mucosal epithelial cell line (GES-1) and gastric cancer cell lines (AGS, BGC-823, SGC-7901). b Transwell assay of the tRF-5026a inhibitor of the normal gastric mucosal epithelial cell line (GES-1) and gastric cancer cell lines (AGS, BGC-823, SGC-7901). Left, representative results. Right, data are presented as the mean ± SD, n=3, nc, negative control, * P<0.05, ** P<0.01, *** P<0.001. [file 13287_2021_2497_MOESM1_ESM.doc]

**Supplementary data**

Supplementary Table S1. Primer sequences for qRT-PCR

| Name | Sequence |
| --- | --- |
| tRF-5026a  RNU6-2 | 5'-TCGGCCGACGATCGTTTCC-3'  5'-CCGCGTCCGATCTCCACTA-3'  5'-GCTTCGGCAGCACATATACTAAAAT-3'  5'-CGCTTCACGAATTTGCGTGTCAT-3' |

Supplementary Table S2. Sensitivity, specificity, AUC of tRF-5026a and tiRNA-5034-GluTTC-2 in tissues and plasma samples and their combination

| Healthy *vs*. Cancer | Sensitivity | Specificity | AUC |
| --- | --- | --- | --- |
| tRF-5026a (tissues) | 0.512 | 0.721 | 0.631 |
| tRF-5026a (plasma) | 0.973 | 0.676 | 0.883 |
| tiRNA-5034-GluTTC-2 (tissues) | 0.733 | 0.465 | 0.779 |
| tiRNA-5034-GluTTC-2 (plasma) | 0.973 | 0.595 | 0.835 |
| tRF-5026a (tissues and plasma) | 0.908 | 0.946 | 0.811 |
| tiRNA-5034-GluTTC-2 (tissues and plasma) | 0.847 | 0.928 | 0.915 |
| tRF-5026a and tiRNA-5034-GluTTC-2 (tissues) | 0.614 | 0.719 | 0.717 |
| tRF-5026a and tiRNA-5034-GluTTC-2 (plasma) | 0.946 | 0.703 | 0.902 |
| tRF-5026a and tiRNA-5034-GluTTC-2 (tissues and plasma) | 0.919 | 0.865 | 0.938 |

Supplementary Table S3. The relationships between the expression levels of tRF-5026a (Δ*C*_q_) in tissues and the clinicopathological factors of patients with gastric cancer.

| Characteristics | *n* (%) | Mean ± SD | *P*-value |
| --- | --- | --- | --- |
| Gender |  |  | 0.879 |
| Male | 66 (76.7) | 13.45 ± 0.5869 |  |
| Female | 20 (23.3) | 13.16 ± 0.7919 |  |
| Age (y) |  |  | 0.143 |
| ≤ 60 | 25 (29.1) | 12.72 ± 0.8257 |  |
| > 60 | 61 (70.9) | 13.81 ± 0.5919 |  |
| CEA |  |  | 0.328 |
| Positive | 80 (93.0) | 13.41 ± 0.4992 |  |
| Negative | 6 (7.0) | 13.98 ± 1.9960 |  |
| CA19-9 |  |  | 0.026 |
| Positive | 59 (68.6) | 12.08 ± 0.5907 |  |
| Negative | 27 (31.4) | 13.49 ± 0.8450 |  |
| Differentiation |  |  | 0.078 |
| Well | 8 (9.3) | 13.93 ± 0.5196 |  |
| Moderate-Poor | 78 (90.7) | 12.65 ± 1.5290 |  |
| Tumor size (cm) |  |  | 0.001 |
| ≤5 | 68 (79.1) | 13.93 ± 0.6511 |  |
| >5 | 18 (20.9) | 12.20 ± 0.7136 |  |
| TNM stage |  |  | 0.132 |
| 0 & I | 14 (16.3) | 13.12 ± 0.5237 |  |
| II | 9 (10.5) | 13.23 ± 0.4254 |  |
| III | 56 (65.1) | 13.18 ± 0.7154 |  |
| IV | 7 (8.1) | 13.85 ± 0.3645 |  |
| Invasion |  |  | 0.524 |
| Tis & T1 | 14 (16.3) | 13.39 ± 1.3241 |  |
| T2 &T3 | 13 (15.1) | 12.98 ± 0.3301 |  |
| T4 | 59 (68.6) | 13.16 ± 0.6505 |  |
| Lymphatic metastasis |  |  | 0.212 |
| N0 & N1 | 41 (47.7) | 13.66 ± 0.5236 |  |
| N2 & N3 | 45 (52.3) | 13.06 ± 0.6173 |  |
| Distal metastasis |  |  | 0.441 |
| M0 | 79 (91.9) | 13.50 ± 0.8255 |  |
| M1 | 7 (8.1) | 13.03 ± 0.4517 |  |

Supplementary Table S4. Univariate and multivariate Cox regression analysis of the overall survival of tRF-5026a expression in patients with gastric cancer tissues.

| Variables | HR | 95% CI | *P* value |
| --- | --- | --- | --- |
| Univariate analysis | | | |
| Gender (Male *vs*. Female) | 5.320 | 0.805-2.692 | 0.019 |
| Age (≤60 *vs*. >60) | 0.495 | 0.174-1.412 | 0.189 |
| CEA (Positive *vs*. Negative) | 2.079 | 0.545-11.548 | 0.283 |
| CA19-9 (Positive *vs*. Negative) | 0.556 | 0.278-2.063 | 0.469 |
| Differentiation (Well *vs*. Moderately + Poorly) | 4.868 | 0.569-9.742 | 0.103 |
| Tumor size (≤5 cm *vs*. >5 cm) | 1.267 | 0.634-6.785 | 0.378 |
| TNM stage (0 + I *vs*. II *vs*. III *vs*. IV) | 3.582 | 1.548-8.285 | 0.003 |
| Invasion depth (Tis + T1 *vs*. T2 + T3 *vs*. T4) | 6.642 | 1.630-15.786 | 0.015 |
| Lymphatic metastasis (N0+ N1 *vs*. N2 *vs*. N3) | 3.128 | 1.345-12.560 | 0.007 |
| Distant metastasis (No *vs*. Yes) | 2.567 | 0.825-13.450 | 0.082 |
| Expression of tRF-5026a in tissue (High *vs*. Low) | 0.678 | 0.138-0.984 | 0.032 |
| Multivariate analysis | | | |
| TNM stage (0 + I *vs*. II *vs*. III *vs*. IV) | 3.694 | 0.347-7.123 | 0.037 |
| Lymphatic metastasis (N0+ N1 *vs*. N2 *vs*. N3) | 3.512 | 1.621-11.128 | 0.046 |


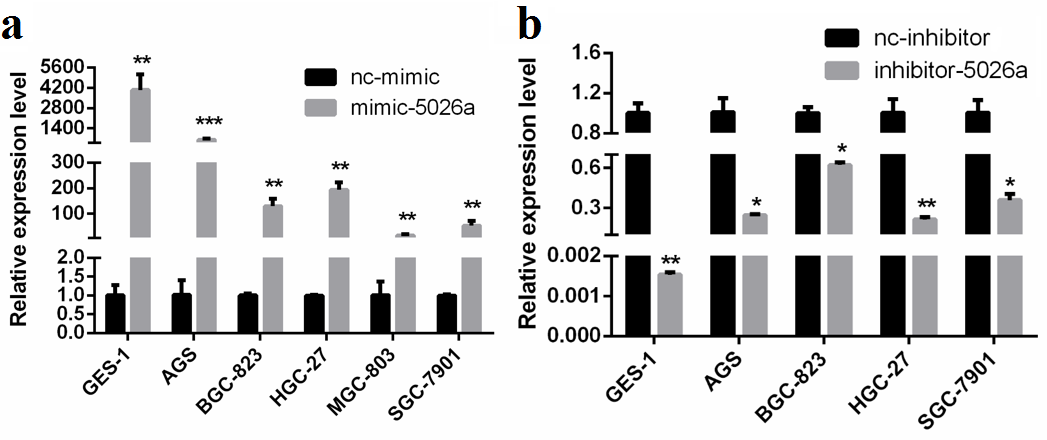


Supplementary Fig. S1 The use of tRF-5026a mimic and inhibitor to modulate its levels in normal gastric mucosal epithelial cells and gastric cancer cells. **a** The up-regulation effect of tRF-5026a mimic. **b** The down-regulation effect of tRF-5026a inhibitor. nc, negative control; *n*=3, * *P*<0.05, ** *P*<0.01, *** *P*<0.001.


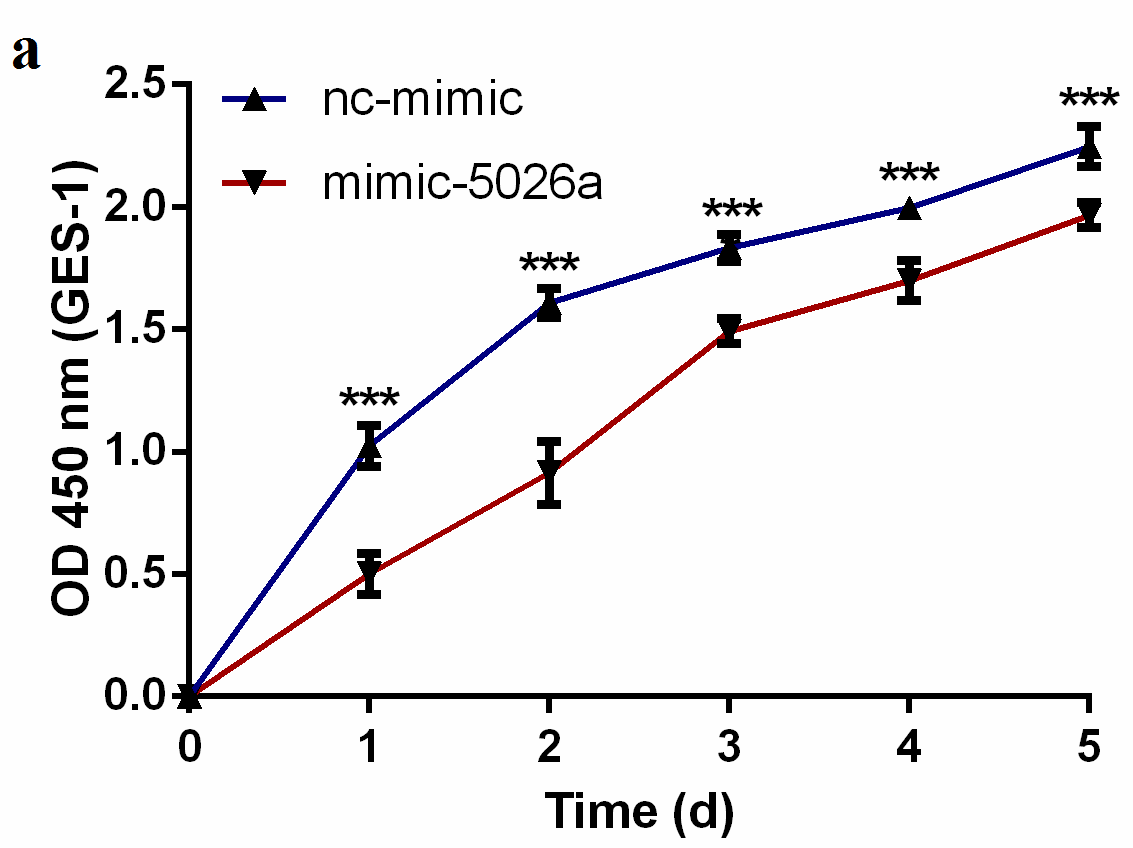

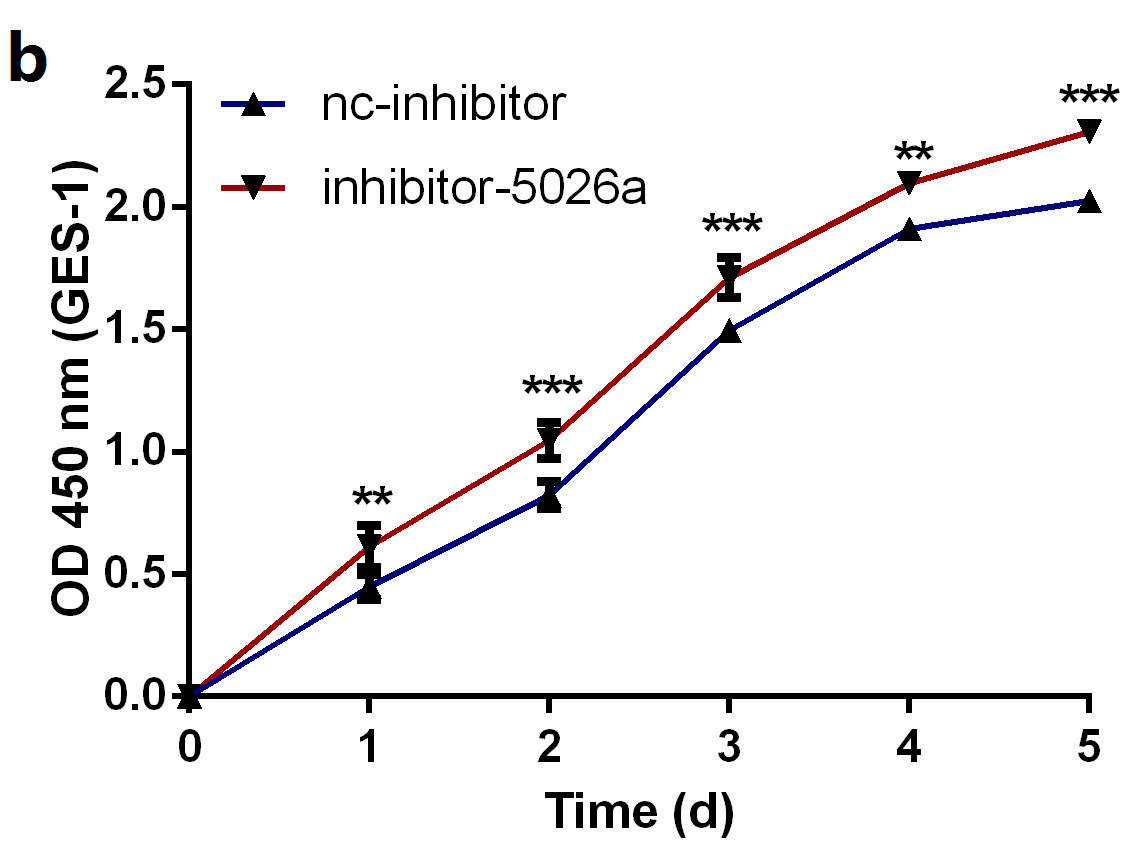


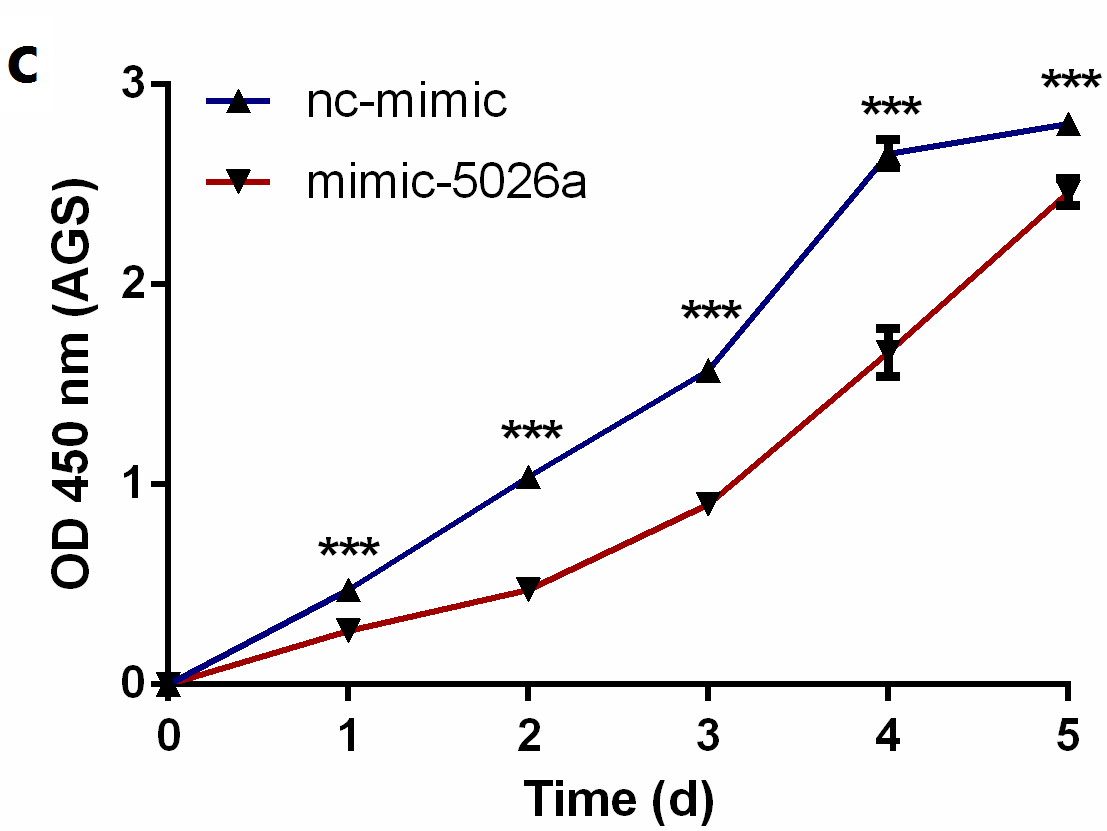

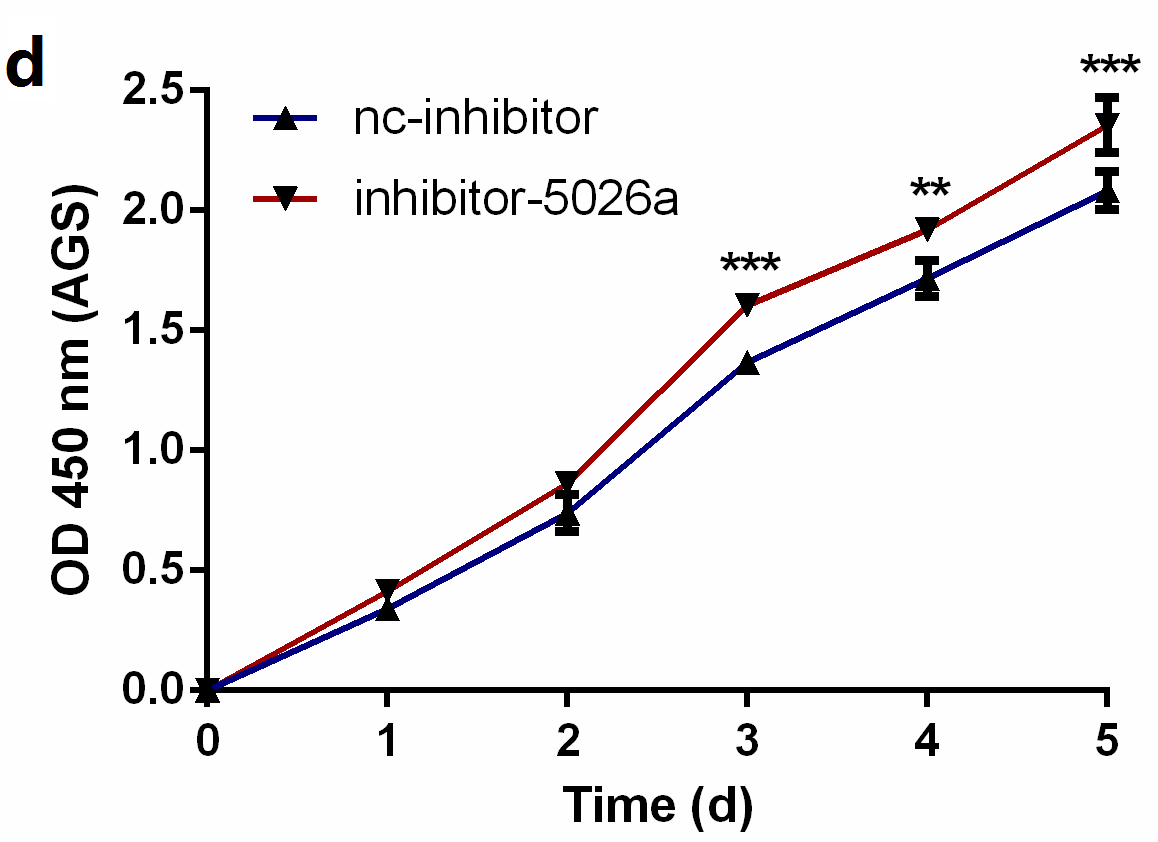


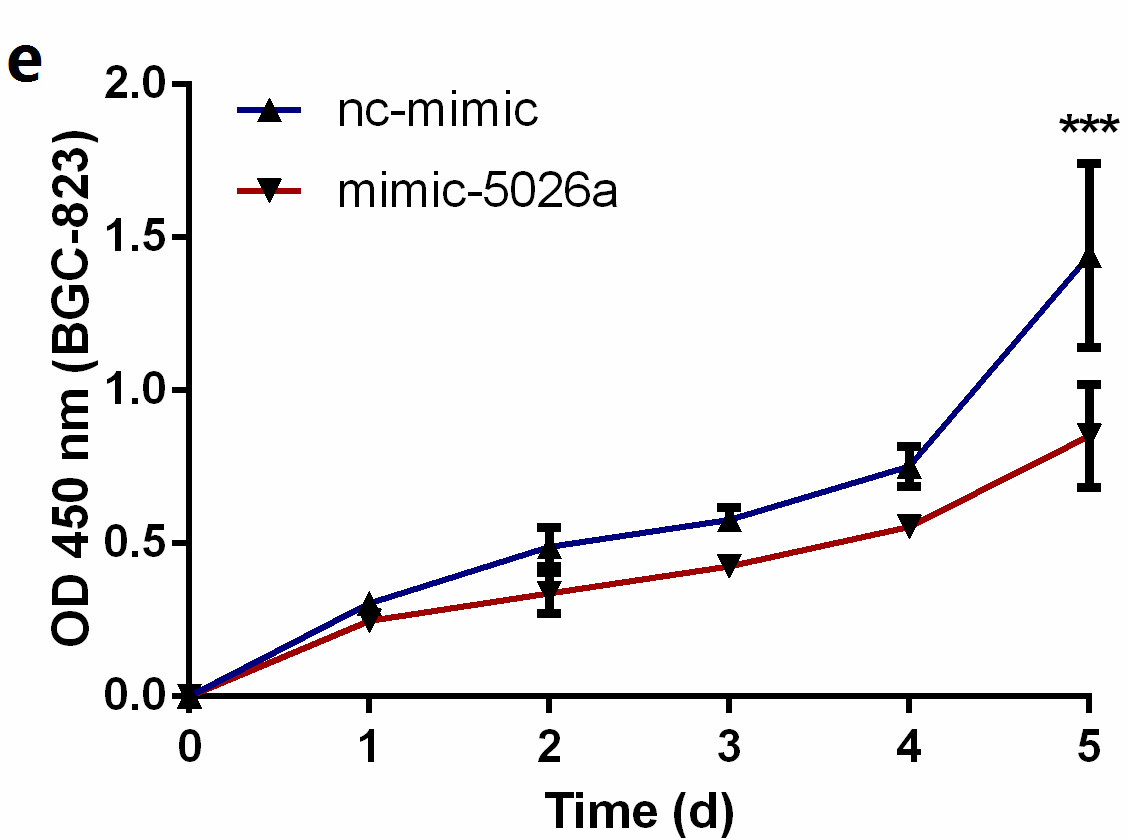

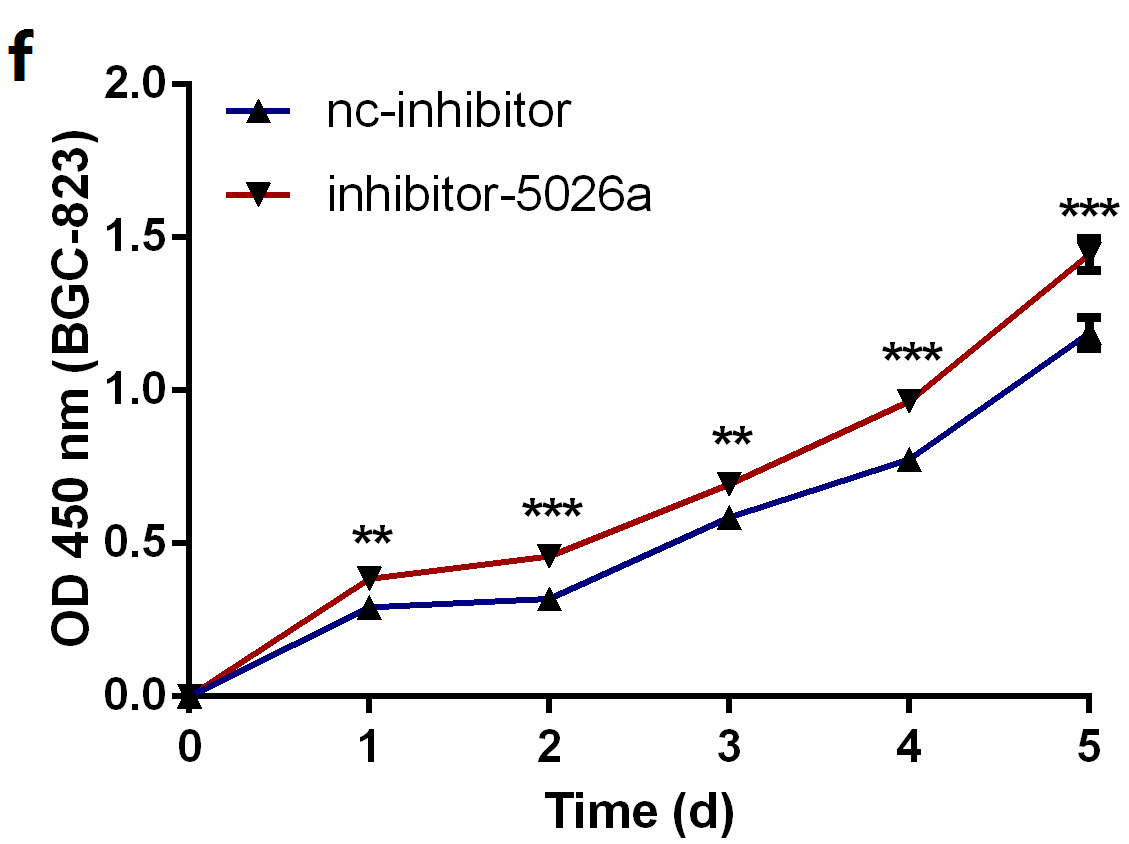


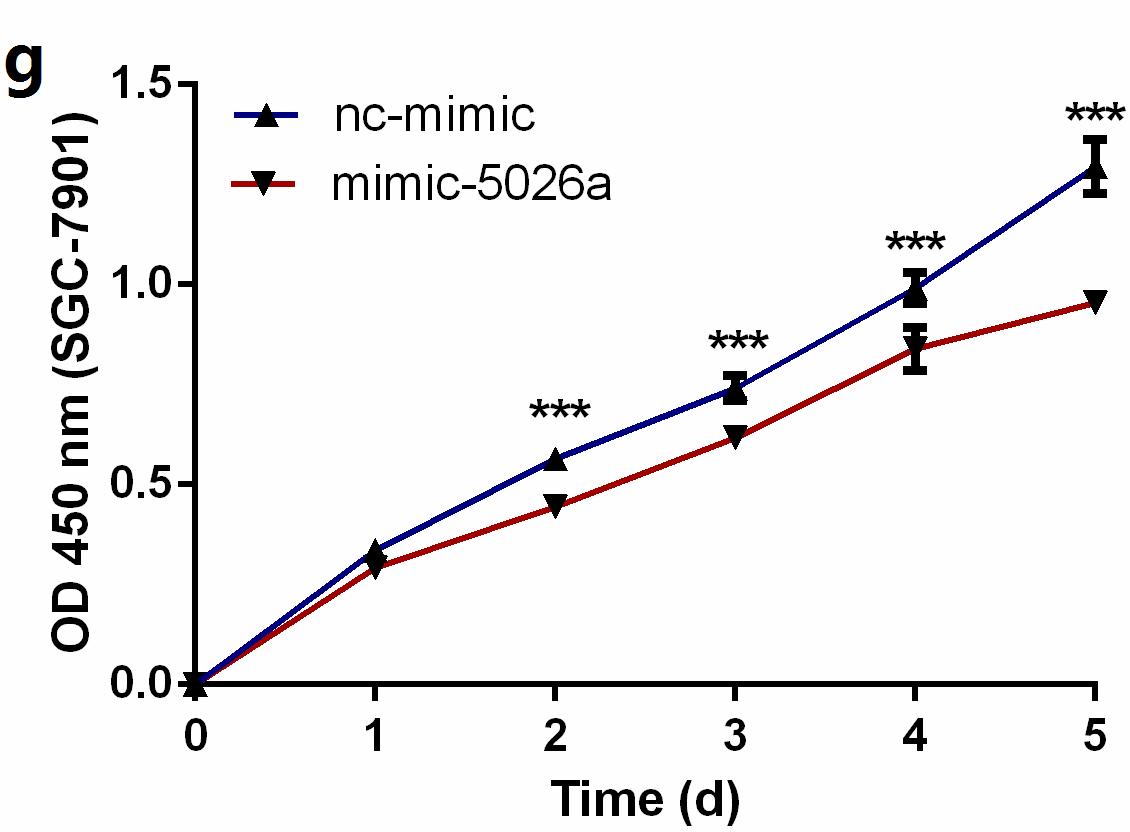

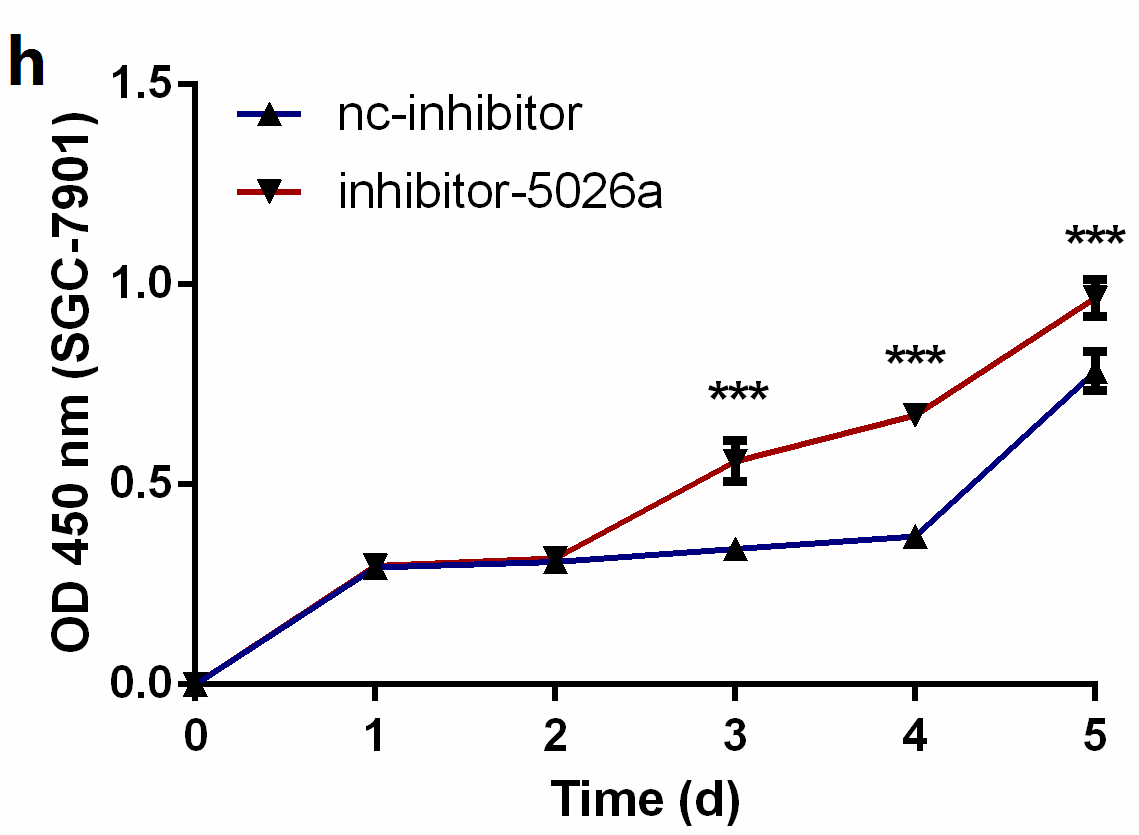


Supplementary Fig. S2 Effects of tRF-5026a on gastric cancer cell proliferation. **a, b** Grow curve of normal gastric mucosal epithelial cell line (GES-1) following up- and down-regulation by tRF-5026a mimic and inhibitor, respectively. **c-h** Grow curve of gastric cancer cell lines (AGS, BGC-823, SGC-7901) following up- and down-regulation by tRF-5026a mimic and inhibitor, respectively. nc, negative control; *n*=6, ** *P*<0.01, *** *P*<0.001.


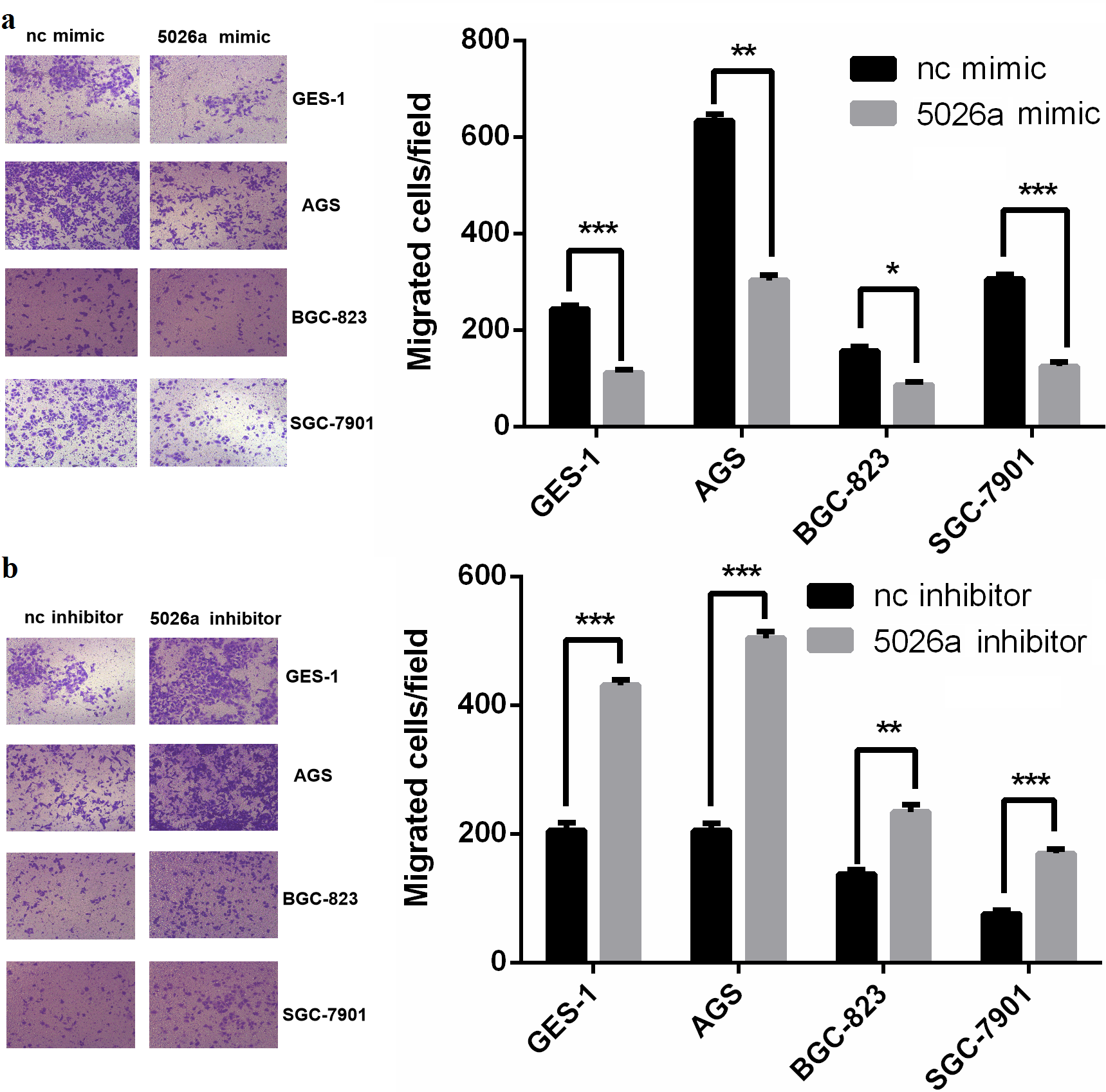


Supplementary Fig. S3 Effects of tRF-5026a on cell migration. **a** Transwell assay of tRF-5026a mimic of the normal gastric mucosal epithelial cell line (GES-1) and gastric cancer cell lines (AGS, BGC-823, SGC-7901). **b** Transwell assay of the tRF-5026a inhibitor of the normal gastric mucosal epithelial cell line (GES-1) and gastric cancer cell lines (AGS, BGC-823, SGC-7901). Left, representative results. Right, data are presented as the mean ± SD, *n*=3, nc, negative control, * *P*<0.05, ** *P*<0.01, *** *P*<0.001.
